# Comparing size selectivity and exploitation pattern of diamond-mesh codends for Southern velvet shrimp (*Metapenaeopsis palmensis*) in shrimp trawl fishery of the South China Sea

**DOI:** 10.7717/peerj.12436

**Published:** 2021-11-02

**Authors:** Bingzhong Yang, Bent Herrmann, Lei Yan, Jie Li, Teng Wang

**Affiliations:** 1 South China Sea Fisheries Research Institute, Chinese Academy of Fishery Sciences, Guangzhou, China; 2 Key Laboratory of Open-Sea Fishery Development, Ministry of Agriculture and Rural Affairs, Guangzhou, China; 3 DTU Aqua, Technical University of Denmark, Hirtshals, Denmark; 4 University of Tromsø, Tromsø, Norway; 5 SINTEF Ocean, Fishing Gear Technology, Hirtshals, Denmark

**Keywords:** Size selectivity, Exploitation pattern, Diamond mesh codend, Southern velvet shrimp, *Metapenaeopsis palmensis*, South China Sea, Trawl, Mesh size, Fisheries management, Shrimp species

## Abstract

In this study, size selectivity and exploitation pattern of six diamond-mesh codends with different mesh sizes, ranging from 25 to 54 mm, for Southern velvet shrimp (*Metapenaeopsis palmensis*) were tested and compared in a shrimp trawl fishery of the South China Sea (SCS). We used a codend with a mesh size of 25 mm (D25) as a starting point, which is the minimum mesh size (MMS) currently regulated in the studied area. Four different fishing population scenarios were applied to quantify and compare how mesh sizes of codends used would impact the size selectivity and exploitation pattern for the target shrimp species. The results demonstrated that the D25 codend was not proper for protecting juvenile shrimp at the studied area. By applying this legal codend, L50 (50% retention length) of the target shrimp species was below its minimum conservation reference size (MCRS, 7.0 cm total length), the retention probability of shrimp with a length of MCRS was above 95% CI [91–99] and more than 43% of undersized shrimp was retained. To mitigate the bycatch issue of undersized shrimp, increasing the mesh size in the diamond mesh codend is a simple and effective option. However, the loss of catch efficiency for marketable shrimp is a major concern while increasing the mesh size. A good compromise between releasing undersized shrimp and maintaining the legal individuals is manifested by using the codend with 35 mm mesh size (D35). Our study will be beneficial for the management of shrimp trawl fisheries in the SCS.

## Introduction

Shrimp trawling is one of the most socio-economically important fisheries, and the annual landing of shrimp was 248,002 t in 2019 from the South China Sea (SCS) (MARA, 2020). The number of commercial vessels associated with shrimp trawling was once reported as 8,500 in this area (Yang, 1992). At present, there is no actual number of shrimp trawlers reported. With the implementation of a vessel buyback program (Cao et al., 2017), it is believed that the number of shrimp trawlers has drastically decreased. To support such a catch volume, however, it has been estimated that the number is still more than 3,000 in the SCS.

The varieties of shrimp species were reported to be more than 350 (Liu & Zhong, 1988), of which about 35 species were economically important in the SCS (Qiu et al., 2008). Among these species, the Southern velvet shrimp (*Metapenaeopsis palmensis*) is one of the most dominant species found in some crustacean resources surveys (Huang et al., 2009a; Huang et al., 2009b). Regardless of species, there are two kinds of fishing gears to target shrimp in commercial fisheries of the SCS. One is the shrimp beam trawl; the other is the double-rigged trawl (Liu & Zhong, 1988). The mouth of the shrimp beam trawl is spread by two beams and a vessel can tow several trawls, often 10 and more, simultaneously (Yang et al., 2018). By comparison, the dimension of the double-rigged trawl is larger both in the fishing circumference and total stretched length. The vertical headline height of a double-rigged trawl is often 1.5 m, and the spread of wing-end is about 15 m during normal fishing. Each vessel can haul two trawls simultaneously using two sets of otterboards. Though differences of gear configuration exist between these two types of shrimp trawls, there are two common points: first, all trawls are made of diamond mesh netting and second, small mesh sizes (often 20 mm or less) used in the codends (Yang, 1992).

Due to small mesh sizes used, a serious bycatch problem was induced in shrimp trawl fisheries of the SCS (Yang et al., 2015; Yang et al., 2017). It was reported that undersized fish and juvenile shrimp constituted the majority of the bycatch species. For instance, Yang et al. (2017) conducted a survey onboard a shrimp beam trawler and reported that more than 90% (in number) of target shrimp were undersized. The bycatch situation did not ameliorate since the implementation of minimum mesh size (MMS) regulation, in which all shrimp trawlers should be required to implement a minimum mesh opening of 25-mm in the codends, in 2014. Recently, some literatures started to question the effectiveness and compliance of this MMS regulation from fisheries management and resource conservation perspectives (Cao et al., 2017; Liang & Pauly, 2017; Zhang et al., 2020). However, very few works have been done to address the bycatch issue from technical consideration, especially from selectivity study. Only two selective experiments have been reported to address the size selectivity of shrimp trawl codends for shrimp species. Zhang, Sun & Luo (2007) estimated the size selectivity of three diamond-mesh codends with mesh sizes of 35, 40 and 45 mm for four penaeid shrimp species (*Metapenaeus ensis*, *M. barbata*, *M. joyneri* and *M. japonicus*). They concluded that the codend with 35 mm mesh size was the best choice to conserve shrimp stock. Later, Yang et al. (2019) conducted a selectivity experiment of beam trawl with two diamond mesh codends, with 25 and 30 mm mesh sizes for greasy-back shrimp (*M. ensis*) and found that selective properties were very poor for these two codends. The conclusions of these two mentioned studies seem to support that the effect of current MMS regulation for shrimp trawl fisheries should be further questioned. Additionally, there is no landing obligation and/or minimum conservation reference size (MCRS) for bycatch and shrimp species implemented in shrimp trawl fisheries to supplement the MMS regulation in the SCS.

It is believed that for a towed fishing gear most of the size selectivity takes place in the codend (Glass, 2000). To improve the size selectivity of a given diamond-mesh codend, the most simple and effective way is to increase the mesh size (Wileman et al., 1996; Fryer, O’Neill & Edridge, 2016; O’Neill et al., 2020). Selectivity of different codends can be quantified by two parameters: L50 (50% retention length) and SR (selection range). Often, modification to gear configurations is aimed to increase the L50 value while decrease the SR or have a constant SR (Kennelly & Broadhurst, 2021). However, previous studies have demonstrated that both L50 and SR would increase with the mesh sizes of codends enlarged (Fryer, O’Neill & Edridge, 2016; O’Neill et al., 2020). Moreover, the exploitation pattern of fishing gears will also be affected by the alteration of mesh size in the codends (Wienbeck et al., 2014). Thus, selective experiments should be conducted to determine which size is optimal to obtain the best size selectivity and exploitation pattern. As mentioned above, few works have been done to address size selectivity in shrimp trawl fishery of the SCS. In particular, no selectivity study associated with the diamond-mesh codends has been reported for the Southern velvet shrimp.

The objective of this study is to address the issues mentioned above. We tested and quantified the size selectivity and exploitation pattern of six diamond mesh codends with different sizes, ranging from 25 to 54 mm, for the Southern velvet shrimp in the commercial fishery, and focused on the following research questions:

1. How did the legal codend with 25-mm mesh size perform for the specific shrimp species?
2. How did the size selectivity and exploitation pattern of codends change with increasing the mesh sizes?
3. If these potential changes were length-dependent?

## Materials & methods

### Sea trials for experimental fishing and population surveys

**Figure 1:**
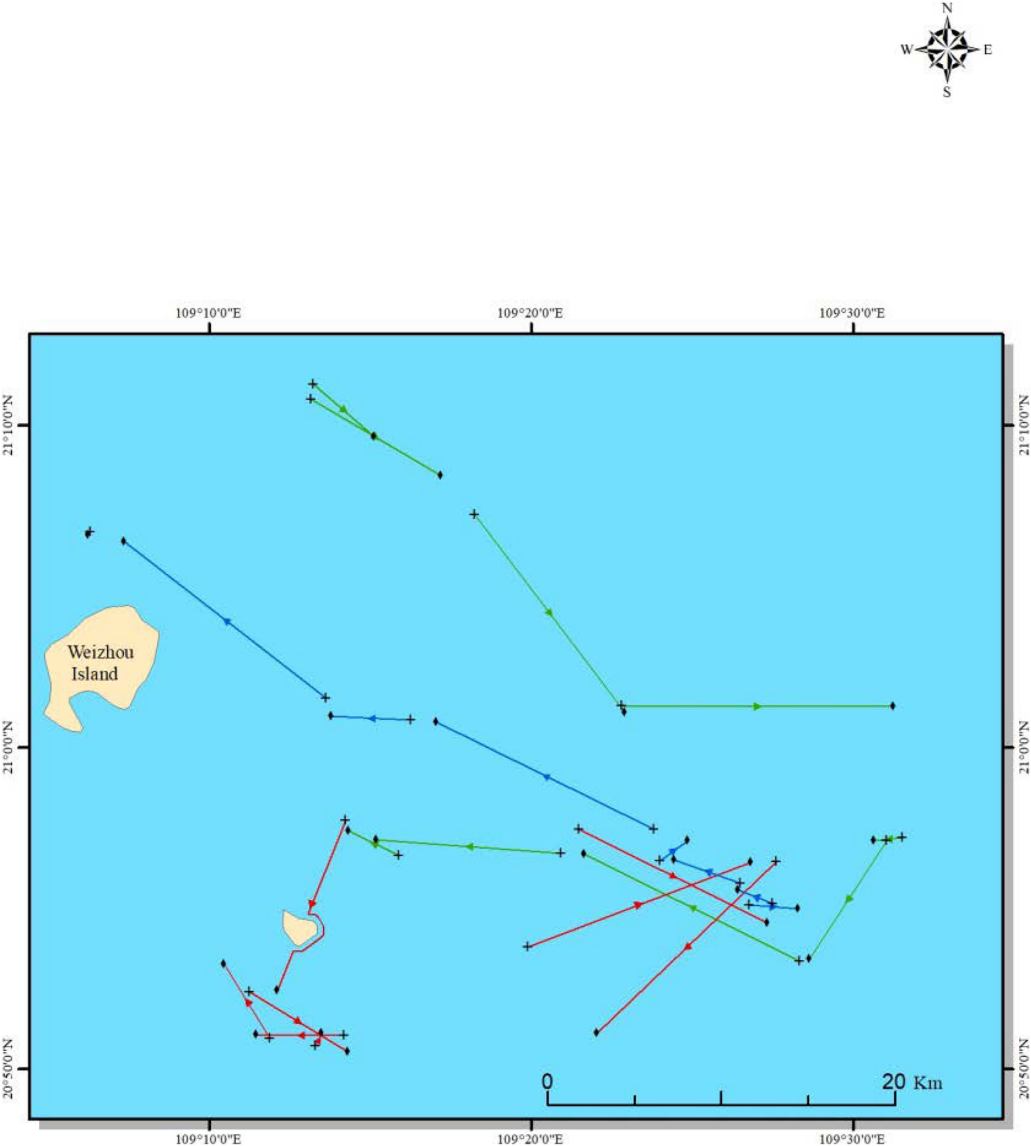
Location of experimental fishing: the colorful lines represent hauling lines. Red lines represent the D25 *vs*. D30 test, purple lines represent the D35 *vs*. D40 test, and green lines represent the D45 *vs*. D54 test, respectively.

Sea trials were carried out onboard a commercial double-rigged shrimp trawler (name ‘*Guibeiyu 96899*’, engine power of 280 kW and total length of 38 m) in October 2019. Experimental fishing was conducted in the Beibu Gulf of the Northern SCS (Fig. 1), where is a traditional area for shrimp trawling and about 37 km from the coastline. To make sure a commercial fishing condition, towing duration and speed were kept mainly at 2 h and 3.5 knot. All experiments have been done during day and night continually, which is typical for the commercial fishery. In addition to the experimental fishing, three population surveys of the target shrimp were conducted onboard the same and similar trawler using the same trawl but with unselective codends in April 2017, November 2018, and March 2019, respectively.

### Fishing gear and experimental set-up

The double-rigged trawls had a 38.7 m fishing circle (860 meshes in circumference with a mesh size of 45 mm) and total stretched length of 32.9 m. These trawls were equipped with a 28 m headline and a 36 m fishing line, to which an 80 m long chain (weighed 210 kg) was attached and acted as a ground-gear. Two sets of rectangle trawl doors (1.6 m^2^ area and 250 kg weight) made of steel and wood were used to spread the trawls.

Fishing gear components were identical with commercial fishery except the codends, which were the main modifications focused in this study. Six diamond mesh codends, mesh sizes ranging from 25 to 54 mm, were designed using the dimension of the commercial codend, which had 220 × 25 mm meshes in the circumference and a stretched length of 4.8 m, as the baseline. The same net maker made all these codends. They were identical in both dimension (circumference and total stretched length) and material; the only differences were the mesh sizes. We termed these codend as D25, D30, D35, D40, D45 and D54, respectively, based on their mesh sizes, and detailed information about the specification and mesh openings were listed in Table 1. Following the recommendation of Wileman et al. (1996), we applied the covered codend method in the experiments. Compared with the experimental codends, the covers used were 1.5 times larger and longer and with a mesh opening about 12 mm (Table 1). To reduce the potential cover-effect, 12 flexible kites were attached around the covers (He, 2007; Grimaldo et al., 2009). Additionally, underwater video recordings (GoPro HERO 4 BLACK Edition) were applied to check whether the cover would mask the tested codend before and during the experimental fishing.

**Table 1:** Overview of specification of the experimental codends and cover.

Codend	Mesh opening ± SD (mm)	Twine diameter ± SD (mm)	Mesh number in circumference	Mesh number in length
D25	25.91 ± 1.05	1.40 ± 0.36	220	192
D30	29.74 ± 0.70	1.24 ± 0.11	183	160
D35	35.70 ± 1.14	1.31 ± 0.10	157	137
D40	40.40 ± 0.85	1.36 ± 0.17	138	120
D45	44.28 ± 0.66	1.24 ± 0.09	122	107
D54	54.54 ± 0.86	1.26 ± 0.09	102	89
Cover	12.51 ± 0.78	1.18 ± 0.10	550	480

**Note:**

SD represents standard errors.

The double-rigged commercial trawler provided us an ideal workplace for the selectivity experiments, in which two separate codends could be tested simultaneously. In order to estimate the effect of mesh sizes on the selectivity of codends, we arranged the pairwised tests as: D25 *vs*. D30, D35 *vs*. D40 and D45 *vs*. D54, respectively. In other words, two pairwised codends were tested at a time for several hauls using the method mentioned above, then moved on to another pairwised test.

During the experimental fishing, all catch of the Southern velvet shrimp from the codend and cover were collected and sampled (if the catch number was huge), and frozen separately after the haul back process. Once we got back to the laboratory, all collected shrimp were counted and length measured to the nearest 1 mm for further selectivity analysis.

### Size-selectivity estimation

The size selectivity of the tested codend for the target shrimp species was carried out separately with the method described below. Our experimental design enabled us to analyze the catch data as binominal data, as the target shrimp was either retained by the cover or the codend in a specific test. The retention probability of a specific codend for a given shrimp with length *l* in haul *j* can be expressed as *r*_*j*_*(l)*. The experimental value of *r*_*j*_*(l)* can be easily calculated by the count number of the codend and the total number (codend + cover); while its theoretical value can be estimated using some parametric models. However, the value of *r*_*j*_*(l)* could be expected to vary for the same codend between different hauls (Fryer, 1991). In order to account for this variation, we estimated the averaged values of *r(l)* by pooling data over all hauls for the tested codends, assuming that size selectivity performance of the tested codend in the experimental fishing can represent how the codend would perform in a commercial fishery (Millar, 1993; Sistiaga et al., 2010; Herrmann et al., 2016; Yang et al., 2021).

The averaged size selectivity mentioned above can be further described as *r*_*av*_*(l)* (Herrmann et al., 2012). Four parametric models, Logit, Probit, Gompertz and Richards (Wileman et al., 1996), were used as candidate to test for *r*_*av*_*(l)*, in which *v* is a vector representing the parameters to be estimated. This analysis is conducted to select the best model to make experimental data most likely to be observed, with the assumption that the experimental data could be described by the selected model sufficiently. We minimized Eq. (1) to estimate the parameter *v*. This analysis was equivalent to maximize the likelihood of the experimental data in the form of number of shrimp, which is the length-dependent, caught by the codend (*nR*_*jl*_) and those by the cover (*nE*_*jl*_):

(1) $$- \sum\limits_{{\rm{j}} = 1}^{\rm{m}} {\sum\limits_{\rm{l}} {\left\{ {{{n{R_{jl}}} \over {q{R_j}}} \times \ln \left( {{r_{av}}\left( {l,v} \right)} \right) + {{n{E_{jl}}} \over {q{E_j}}} \times \ln \left( {1.0 - {r_{av}}\left( {l,v} \right)} \right)} \right\}} }$$

where the outer summation is over the *m* hauls carried out, while the inner summation is over length class *l*; *qR*_*j*_ and *qE*_*j*_ is the sub-sampling rate of the shrimp species length measured from the codend and cover, respectively.

A two-step procedure was applied: first, each of the candidate models was initially fitted in Eq. (1), then selected the best model with the lowest Akaike’s information criterion (AIC) values (Akaike, 1974); second, once the best model was identified for a given codend, a double bootstrapping technique was applied to calculate the Efron percentile 95% (Efron, 1982) confidence intervals (CIs) for the size selectivity and parameters, incorporating both within- and between-haul variation (Millar, 1993; Herrmann et al., 2012; Yang et al., 2021). Finally, the ability of the selected model to describe the experimental data sufficiently well can be evaluated according to the *p*-value, which represents the likelihood of obtaining at least as big a discrepancy between the modelled result and the observed data. Generally, the *p*-value from the selected model should not be less than 0.05; if it is not, *p*-value < 0.05, we should inspect the residuals to check whether the low *p*-value was due to structural problems of the model to represent the experimental data or if it was simply due to overdispersion in the data (Wileman et al., 1996; Yang et al., 2021).

### Delta selectivity

In order to explore how the size selectivity of tested codends for the target shrimp change with increasing mesh sizes, we calculated delta selectivity, Δ*r*(*l*), by the following expression:

(2) $$\Delta r(l) = r_{2}(l) - r_{1}(l)$$

where *r*_*2*_ (*l*) is the size selectivity for codend 2 with a larger mesh size, and *r*_*1*_(*l*) represents the size selectivity for codend 1 with a smaller mesh size. The Efron 95% CIs of Δ*r*(*l*) was calculated according to the two bootstrap populations of results for both *r*_*1*_(*l*) and *r*_*2*_(*l*). Because they were obtained independently, we created a new bootstrap population for Δ*r*(*l*) by the following expression:

(3) $$\Delta r (l)_{i} = r_{2}(l)_{i} - r_{1}(l)_{i} i \in [1 \ldots1000]$$

where *i* is the bootstrap repetition index. As the bootstrap re-sampling of the two populations was random and independent, it is valid to generate the bootstrap population of results for the difference based on Eq. (3) (Herrmann, Krag & Krafft, 2018; Herrmann et al., 2019; Yang et al., 2021).

### Estimation of exploitation pattern indicators

In addition to the estimation of size selectivity, it is essential to quantify how the tested codends with different mesh sizes perform under the same fishing population of the target shrimp, a specific scenario of population, *nPop*_*l*_, was generated by pooling length data from both cover and coded over all hauls (Melli et al., 2019; Einarsson et al., 2021; Yang et al., 2021). In total, four different fishing population scenarios of the target shrimp species were generated. Applying the size selectivity predicted in the previous section, four exploitation pattern indicators, *nP−*, *nP*+, *nRatio*, and *dnRatio* (Eq. (4)), were calculated for each codend with a MCRS of the target shrimp. Specifically, as there is no formal MCRS promulgated for the Southern velvet shrimp in the studied area, we used its first matured total length of 7.0 cm (Liu & Zhong, 1988) as a MCRS to calculate the indicators.

(4) $$\matrix{ {\matrix{ {\matrix{ {nP - = 100 \times {{\sum\limits_{l \lt MCRS} {\left\{ {{r_{codend}}\left( l \right) \times nPo{p_l}\} } \right.} } \over {\sum\limits_{l \lt MCRS} {\left\{ {nPo{p_l}\} } \right.} }}} \cr {nP + = 100 \times {{\sum\limits_{l \ge MCRS} {\left\{ {{r_{codend}}\left( l \right) \times nPo{p_l}\} } \right.} } \over {\sum\limits_{l \ge MCRS} {\left\{ {nPo{p_l}\} } \right.} }}} \cr {nRatio = {{\sum\limits_{l \lt MCRS} {\left\{ {{r_{codend}}\left( l \right) \times nPo{p_l}\} } \right.} } \over {\sum\limits_{l \ge MCRS} {\left\{ {{r_{codend}}\left( l \right) \times nPo{p_l}\} } \right.} }}} \cr } } \cr {dnRatio = 100 \times {{\sum\limits_{l \lt MCRS} {\left\{ {{r_{codend}}\left( l \right) \times nPo{p_l}\} } \right.} } \over {\sum\limits_l {\left\{ {{r_{codend}}\left( l \right) \times nPo{p_l}\} } \right.} }}} \cr } } \cr }$$

where *r*_*codend*_*(l)* is the size selectivity from the tested codend, and *nPop*_*l*_ is the size structure of fishing shrimp population with length class *l*. The indicator *nP−* and *nP*+ is the percentage of shrimp with length below and above the MCRS retained by the tested codend, respectively. For a tested codend with good selective properties, *nP−* value is expected to be close to 0 while *nP*+ value close to 100%. The indicator *nRatio* represents the landing ratio of shrimp with length below and above the MCRS. The indicator *dnRatio* is the total percentage of shrimp with length below the MCRS retained by the tested codend. To have good selective properties, *nRatio* and *dnRatio* for the tested codends is expected to be the lower the better. Again, we used the double bootstrapping approach mentioned above to estimate the Efron percentile 95% CIs for the indicator values.

The data analysis mentioned above, including size-selectivity estimation, delta selectivity and estimation of exploitation pattern indicators were conducted using the selectivity software SELNET (Herrmann et al., 2012).

## Results

### Experimental and survey data

In total, 47 valid hauls were carried out; eight hauls for each codend, except the D45 codend for which seven hauls were conducted (Table 2). The averaged duration was about 130 min, with a range of 118 to 156 min, and the water depth was mainly 16 m, ranging from 12 to 24 m, in the fishing grounds. Among the species caught, Southern velvet shrimp was present in all hauls and was the most dominant species in terms of quantity. We obtained sufficient number of the target shrimp to be included in selective analysis, 3,334 in total, 1,815 individuals from the tested codends and 1,519 from the covers. Sub-sampled ratios for the target species ranged from 0.2 to 1.0, depending on the catch amount of the specific haul.

**Table 2:** Overview of the hauls in the data analysis.

Codend	Haul no.	Duration (min)	Depth (m)	*nR*	*qR*	*nE*	*qE*
D25	1	124	18	37	0.50	9	1.00
D25	2	119	19	83	0.50	27	1.00
D25	3	118	19	65	0.50	8	1.00
D25	4	130	18	33	0.50	0	1.00
D25	5	156	18	100	1.00	30	1.00
D25	6	127	17	35	0.50	54	1.00
D25	7	130	14	65	0.50	43	1.00
D25	8	140	15	37	0.50	87	1.00
D30	1	124	18	46	0.50	6	1.00
D30	2	119	19	74	0.50	4	1.00
D30	3	118	19	51	0.50	27	1.00
D30	4	130	18	76	0.50	31	1.00
D30	5	156	18	38	1.00	19	1.00
D30	6	127	17	20	0.50	16	0.50
D30	7	130	14	24	0.50	80	1.00
D30	8	140	15	33	0.50	50	1.00
D35	1	128	17	58	0.50	0	1.00
D35	2	135	17	30	1.00	30	1.00
D35	3	149	17	42	0.50	32	0.50
D35	4	153	16	60	0.50	70	0.50
D35	6	154	12	0	1.00	100	0.50
D35	7	134	13	37	0.50	25	0.33
D35	8	130	15	26	1.00	17	1.00
D35	9	122	17	3	1.00	14	0.50
D40	1	128	17	38	0.50	54	1.00
D40	2	135	17	15	1.00	27	1.00
D40	3	149	17	32	0.50	12	0.50
D40	4	153	16	114	0.50	56	0.50
D40	5	143	12	6	1.00	126	0.33
D40	7	134	13	31	1.00	61	0.25
D40	8	130	15	45	1.00	28	0.50
D40	9	122	17	9	1.00	10	0.33
D45	1	125	13	18	1.00	17	0.33
D45	2	128	12	16	1.00	48	0.33
D45	3	120	13	55	1.00	46	0.25
D45	5	122	17	58	0.50	51	0.33
D45	6	124	17	30	0.50	9	0.33
D45	7	122	17	15	1.00	24	0.33
D45	8	124	24	0	1.00	5	0.33
D54	1	125	13	0	1.00	18	0.25
D54	2	128	12	99	1.00	20	0.20
D54	3	120	13	35	1.00	17	0.20
D54	4	122	13	43	1.00	42	0.20
D54	5	122	17	49	0.50	19	0.33
D54	6	124	17	17	0.50	37	0.33
D54	7	122	17	4	0.50	13	0.25
D54	8	124	24	13	1.00	0	0.25

**Note:**

Haul number, duration (min), depth (m), and the number of length measurement from the codend (*nR*), and cover (*nE*), while *qR* and *qE* represents the sampling ratio from the codend and cover, respectively.

**Figure 2:**
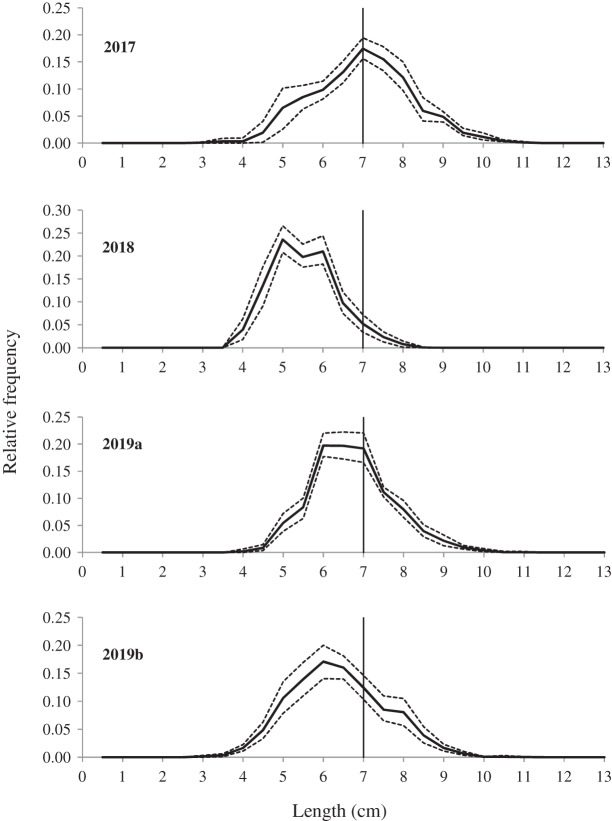
Estimated average population from different fishing population scenarios. The first three curves (2017, 2018 and 2019a) represent fishing population scenarios obtained from surveys, while the last curve (2019b) is the population found in the experimental fishing. Stipple lines show the 95% Efron confidence intervals, and the vertical lines represent the MCRS (minimum conservation reference size) of the Southern velvet shrimp.

The fishing population scenarios were generated by pooling length distribution of all Southern velvet shrimp in four different sea trials (Fig. 2). These population scenarios were based on sufficient number of length measurement for the target shrimp. In addition to the number of individuals in the experimental fishing mentioned above, there were 2,632, 1,726 and 1,860 individuals of length measurement in the population survey of 2017, 2018 and 2019, respectively.

### Size selectivity

Based on the AIC values of four candidate models, the best model was selected as: the Gompertz for the D25 codend, the Richards for the D30 codend, the Probit for the D35 and D40 codend, and the Logit for the D45 and D54 codend, respectively (Table 3).

**Table 3:** Akaike’s information criterion (AIC) for each model of the tested codends.

Codend	Models			
	Logit	Probit	Gompertz	Richards
D25	556.09	580.69	**550.79**	551.93
D30	497.29	499.84	496.91	**496.80**
D35	1,137.27	**1,135.40**	1,141.30	1,138.36
D40	1,491.27	**1,489.18**	1,490.49	1,491.63
D45	**993.52**	993.87	997.43	995.46
D54	**1,205.22**	1,205.90	1,206.11	1,207.13

**Note:**

Selected model in bold.

**Figure 3:**
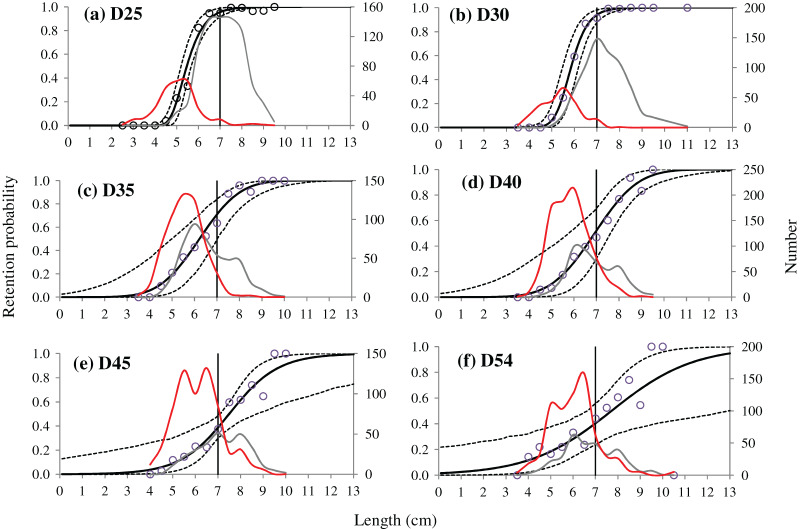
(A–F) Experimental catch proportion and fitted selection curves. Circle marks represent experimental catch proportion. Red curves represent the size distribution of shrimp caught by the cover, grey curves represent the one caught by the codend. Stippled curves describe the 95% confidence intervals for the fitted selection curves. Vertical lines represent the MCRS (minimum conservation reference size) of the target species.

The fit statistical results demonstrated that all selected models were able to express the experimental data well, except the D25 codend which a *p*-value < 0.05 was obtained. For this codend, however, as the selectivity curve represented the main trend of the experimental data well (Fig. 3A), we concluded that this low *p*-value was due to overdispersion in the fishing data.

As the mesh sizes increased, the selective parameters of the tested codends, both L50 and SR, for the target species represented an increasing trend. For instance, L50 and SR of the D25 codend was 5.47 and 0.89 cm, respectively, whereas the relative value increased to 7.72 and 4.01 cm for the D54 codend, respectively (Table 4). The codends with the largest mesh sizes, D45 and D54, had significantly larger L50 than the codends with smaller mesh sizes, the D25, D30 and D35 codend, respectively, while the SR of the D45 and D54 codend was significantly larger than those of the D25 and D30 codend, respectively. Additionally, the CIs in selective parameters became wider for the tested codends as the mesh sizes increased, especially for the D54 codend. The results from selectivity curves showed a similar fishing pattern. As the mesh sizes of tested codends increased, the retention probability for shrimp with a MCRS length decreased. For example, the retention probability of shrimp with a MCRS length in the D25 codend was larger than 95%, while this value dropped to 52% for the D40 codend and below 41% for the D45 and D54 codend, respectively.

**Table 4:** Selective parameters and fit statistics obtained from the selected models for the tested codends.

	Codends					
Parameters	D25	D30	D35	D40	D45	D54
Model	Gompertz	Richards	Probit	Probit	Logit	Logit
L50 (cm)	5.47 (5.20–5.67)	5.85 (5.47–6.18)	6.22 (4.99–7.01)	6.92 (5.77–7.62)	7.49 (7.09–8.71)	7.72 (6.63–12.93)
SR (cm)	0.89 (0.62–1.14)	0.81 (0.61–1.04)	1.90 (1.37–3.47)	2.05 (1.38–4.21)	2.37 (1.45–9.36)	4.01 (1.96–19.68)
δ		2.51 (0.61–10.00)				
*p*-value	0.0069	0.9501	0.72	0.8328	0.7109	0.0690
Deviance	28.84	4.57	8.77	6.57	8.03	21.20
DOF	13	11	12	11	11	13

### Delta selectivity

**Figure 4:**
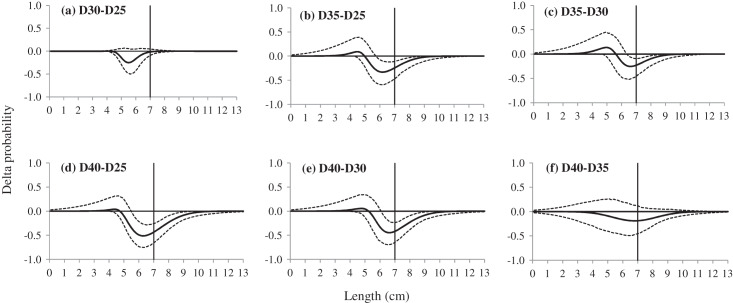
(A–F) Delta selectivity curves of comparison between four codends, the D25, D30, D35 and D40 codend. The solid black curves represent the delta selectivity for each comparison, and the stippled curves represent the 95% confidence intervals. Vertical lines represent the MCRS (minimum conservation reference size) of the target species.

**Figure 5:**
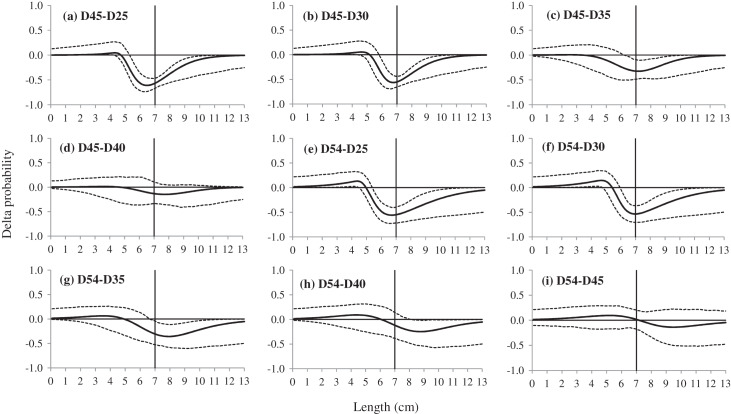
(A–F) Delta selectivity curves of comparison between six codends, the D25, D30, D35, D40, D45 and D54 codend. The solid black curves represent the delta selectivity for each comparison, and the stippled curves represent the 95% confidence intervals. Vertical lines represent the MCRS (minimum conservation reference size) of the target species.

The results of delta selectivity demonstrated that applying codends with larger mesh sizes would decrease the retention probability for the studied species, and most of these differences were statistically significant and length-dependent. For instance, comparing with the D25 and D30 codend, the D35 and D40 codend had significant lower retention probability for shrimp with an approximate length range of 5.5 to 10.0 cm (Fig. 4). The D45 codend significantly retained fewer shrimp with lengths above 5.4 cm, 5.8 cm and 6.3 cm than the D25, D30 and D35 codend, respectively (Fig. 5). A similar tendency was obtained for the D54 codend by comparison with the codends with smaller mesh sizes. The effect of four comparisons was not significant including D30 *vs*. D25, D40 *vs*. D35, D45 *vs*. D40 and D54 *vs*. D45, respectively.

### Exploitation pattern indicators

The exploitation pattern indicators showed that catch efficiency, both undersized (*nP−*) and marketable size (*nP+*), of codends used for the target shrimp decreased with the mesh sizes increased. In all fishing population scenarios, for instance, the D25 codend retained more than 43% of undersized shrimp (*nP−*), the ratios were above 29% for the D30 and D35 codend, respectively, whereas the percentages dropped to less than 29% when the mesh sizes used larger than 35 mm (Table 5). Applying codends with larger mesh sizes, however, would compromise the catch efficiency of shrimp with marketable sizes. For example, the D25 and D30 codend retained more than 95% of legal size shrimp (*nP+*), and the D35 codend retained above 75%. In comparison, about 60% of shrimp with marketable size was caught by the D40 codend, and the ratios were nearly 50% for the D45 and D54 codend. Some of these differences mentioned above were statistically significant. The results of *nRatio* and *dnRatio* varied in different fishing population scenarios for the same codend, and differences between codends were not statistically significant in the same scenarios. For instance, the discard percentage of shrimp caught (*dnRatio*) was below 30% for population scenario in 2017, the relative values were all above 76% for all codends used in population scenario of 2018.

**Table 5:** Performance indicators obtained for the tested codends.

Codend	Population	*nP−* (%)	*nP+* (%)	*nRatio*	*dnRatio* (%)
D25	2017	61.69 (50.68–77.12)	98.01 (95.78–99.55)	0.43 (0.33–0.54)	29.99 (24.65–35.13)
	2018	43.83 (34.22–55.71)	96.65 (93.35–99.18)	4.93 (3.45–7.89)	83.13 (77.53–88.75)
	2019a	70.31 (62.25–81.90)	97.51 (94.80–99.40)	0.85 (0.68–1.05)	46.09 (40.62–51.31)
	2019b	57.13 (48.13–69.22)	97.77 (95.23–99.47)	1.07 (0.76–1.55)	51.63 (43.16–60.74)
D30	2017	47.99 (32.96–66.67)	97.54 (94.27–99.32)	0.33 (0.23–0.45)	25.09 (18.64–31.23)
	2018	30.00 (18.15–45.01)	95.66 (90.40–98.72)	3.41 (2.02–5.99)	77.32 (66.91–85.69)
	2019a	56.68 (40.59–72.30)	96.85 (93.00–99.05)	0.69 (0.49–0.94)	40.97 (32.78–48.48)
	2019b	43.30 (29.81–58.82)	97.22 (93.59–99.18)	0.81 (0.51–1.29)	44.86 (33.64–56.42)
D35	2017	39.39 (18.34–63.79)	83.88 (68.99–91.88)	0.32 (0.17–0.52)	24.22 (14.44–34.22)
	2018	29.65 (11.63–55.84)	75.98 (56.51–86.99)	4.24 (2.16–8.74)	80.92(68.40–89.73)
	2019a	43.74 (21.50–66.20)	81.04 (64.35–89.84)	0.64 (0.37–0.97)	39.02 (27.10–49.24)
	2019b	36.68 (17.08–61.03)	82.39 (66.63–90.84)	0.81 (0.42–1.47)	44.86 (29.72–59.52)
D40	2017	24.86 (8.25–50.73)	69.53 (54.24–82.37)	0.24 (0.09–0.47)	19.57 (8.56–32.05)
	2018	17.79 (4.63–44.82)	58.19 (38.24–74.14)	3.32 (1.18–8.40)	76.87 (54.16–89.36)
	2019a	27.92 (9.80–52.87)	65.42 (47.68–79.29)	0.51 (0.22–0.88)	33.60 (18.31–46.74)
	2019b	22.87 (7.13–48.68)	67.16 (50.48–80.72)	0.62 (0.22–1.35)	38.36 (18.28–57.53)
D45	2017	18.70 (9.64–36.64)	56.07 (42.15–65.06)	0.23 (0.11–0.53)	18.50 (9.99–34.50)
	2018	13.94 (6.16–33.49)	44.34 (34.28–53.28)	3.42 (1.54–9.13)	77.36 (60.62–90.13)
	2019a	20.74 (11.30–38.08)	51.78 (39.28–60.20)	0.47 (0.26–0.99)	32.20 (20.36–49.74)
	2019b	17.36 (8.86–35.58)	53.42 (40.02–61.90)	0.59 (0.28–1.55)	37.25 (21.72–60.82)
D54	2017	26.41 (13.28–42.34)	51.17 (31.69–67.79)	0.35 (0.19–0.60)	26.00 (15.75–37.67)
	2018	22.43 (9.99–39.42)	43.64 (26.18–59.40)	5.59 (2.75–11.57)	84.82 (73.31–92.05)
	2019a	28.19 (14.87–44.24)	48.37 (29.53–64.39)	0.69 (0.40–1.09)	40.86 (28.48–52.13)
	2019b	25.29 (12.39–41.44)	49.32 (30.25–65.47)	0.94 (0.45–1.82)	48.36 (31.07–64.52)

**Note:**

The first three populations (2017, 2018 and 2019a) were obtained from surveys, while the last population (2019b) was from the experimental fishing.

## Discussion

In this study, we tested and compared the size selectivity and exploitation patterns of diamond mesh codends, with a large mesh size range, in a shrimp trawl fishery of the SCS. In particular, our results demonstrated that size selectivity and exploitation pattern of codends for the target shrimp could be modified by simply increasing mesh sizes. The approaches applied in our study not only enable us to quantify the absolute size selectivity of codends for the specific species, but also to estimate how the exploitation pattern would be under different fishing population scenarios.

First of all, our results demonstrated that the selective properties of the legal D25 codend were not satisfactory for the need of protecting juvenile shrimp of the studied area. Applying this legal codend resulted in a L50 far below the MCRS of the specific shrimp species, the retention probability of shrimp with a length of MCRS was above 95% CI [91–99] and more than 43% of undersized shrimp would be retained. The implication of all these was that a serious bycatch issue of undersized shrimp would be induced if the current MMS regulation does not modify.

By comparing the size selectivity and exploitation pattern of codends with different mesh sizes, our study can provide a direction for gear modification in shrimp trawl fishery of the SCS. To mitigate the bycatch issue of undersized shrimp, increasing the mesh sizes in the diamond mesh codend is a simple and effective choice. Comparing with the D25 codend, codends with larger mesh sizes (*e.g.*, the D35 or D40 codend) would have higher L50 values, the retention probability of shrimp at the MCRS length and catch ratio of undersized shrimp would all decrease. However, the mesh sizes in diamond mesh codends were not the larger the better. The largest disadvantage is the compromised loss of shrimp with marketable sizes. For instance, applying codends with the largest mesh sizes, the D45 and D54 codend, *nP+* values were nearly 50%, implying that half of the shrimp with legal sizes would escape from the codends. This is unacceptable from the economic perspective of fishermen. Additionally, higher SR values were obtained when the mesh sizes of codends were above 40 mm, and the CIs in the selective parameters became wider. With the consideration of releasing undersized shrimp and maintaining catch efficiency of the legal one, the D35 codend would be the best choice. Because it obtained a L50 close to the MCRS of the Southern velvet shrimp, 71% of retention probability for shrimp at the MCRS length, and less than 44% of juvenile shrimp and more than 75% of legal shrimp would be retained. Moreover, when the mesh size was further enlarged to 40 mm (the D40 codend), for instance, the effect of releasing undersized shrimp was not statistically significant (Fig. 4).

To the best of our knowledge, there is no selectivity study associated with trawl codends for the Southern velvet shrimp reported in the SCS. Though no literature can be compared with our results, an earlier study conducted by Zhang, Sun & Luo (2007) using three diamond mesh codends, with mesh sizes of 35, 40 and 45 mm, respectively, to select four different shrimp species, their results showed that the codend with 35 mm was an appropriate choice to protect juvenile shrimp. It might be hard to compare their result with ours because different shrimp species were focused on. But the conclusion is nearly identical that the 35-mm diamond mesh codend had good selective properties to target shrimp species.

It is widely accepted that size selectivity is not only affected the configuration of fishing gears, but also by the morphology, swimming capacity and behaviour of the target species (Wileman et al., 1996; Herrmann et al., 2009). To better understanding the size selectivity of shrimp species, it is also important to estimate how the shrimp would contact the fishing gears (*e.g.*, codend netting) (Brčić et al., 2018). At present, there is no study addressing the swimming and behaviour of the Southern velvet shrimp reported in the literature. But our underwater recordings showed that direction of the studied species escaping from the codend netting varied between individuals. The implication was that contact behaviour of the studied species in the codends might vary and contribute to some uncertainties to selective properties, especially for the codends with larger mesh sizes.

To improve size selectivity, mesh size is an important modification to be considered. But it is not the only one. Other modifications, such as mesh shape, codend circumference and twine diameter, have been tested and proved to have effect on size selectivity (Wileman et al., 1996; Santos et al., 2018; Kennelly & Broadhurst, 2021). Future research work for improving size selectivity of the Southern velvet shrimp should take these modifications into account.

## Conclusions

To summarize, our study demonstrated that the legal D25 codend was not proper for protecting juvenile shrimp at the studied area and the size selectivity and exploitation pattern of codends for the target shrimp could be improved by simply increasing the mesh sizes. The D35 codend will be an appropriate choice to target this species.

## Supplemental Information

10.7717/peerj.12436/supp-1 Supplemental Information 1 Raw data of length measurements of the target species. 2017, 2018 and 2019a were from fishery surveys, while the last 2019b data were from the selectivity experiments.

## Additional Information and Declarations

### Data Availability

The following information was supplied regarding data availability: The raw data are available in the Supplementary File.
